# Enhanced photocatalytic degradation of sunset yellow dye using a novel SrFe_12_O_19_/PANI/graphene nanoplatelets ternary nanocomposite under visible light and visible light/H_2_O_2_ irradiation

**DOI:** 10.1039/d6ra04115a

**Published:** 2026-07-06

**Authors:** Ammar Ul Haq, Muhammad Tariq, Haseeb Ahmed Tajik, Muhammad Asim Safi, Muthanna K. Kareem, Wilayat Khan, Tanveer Ul Haq Zia

**Affiliations:** a National Centre of Excellence in Physical Chemistry, University of Peshawar 25210 Pakistan; b Department of Physical and Numerical Sciences, Qurtuba University of Science and Information Technology Peshawar Pakistan; c Department of Physics, University of Peshawar 25210 Pakistan asimsafi.scientist@gmail.com; d College of Engineering, Al-Ayen University Thi-Qar Iraq; e Department of Physics, Bacha Khan University Charsadda Pakistan; f Institute of Chemical Sciences, University of Peshawar 25210 Pakistan

## Abstract

Durable and highly active photocatalysts are critically needed for the treatment of persistent synthetic azo dyes in wastewater. Herein, a novel ternary SrFe_12_O_19_/PANI/GnPs (SPG) nanocomposite was synthesized *via* the *in situ* chemical oxidative polymerization method for the photocatalytic degradation of the anionic sunset yellow dye under visible light and visible-light/H_2_O_2_ irradiation. Structural and optical analyses confirmed the effective integration of all three materials, leading to enhanced light absorption and improved charge separation. XRD analysis verified the preservation of the hexaferrite magnetoplumbite structure, alongside the characteristic graphene reflection and an amorphous polyaniline hump. FTIR analysis further validated the hexaferrite lattice integrity, while SEM-EDX revealed homogeneous PANI/GnPs embedded within the SrFe_12_O_19_ matrix. UV-vis analysis demonstrated a significant red shift in light absorption upon ternary nanocomposite formation, with the bandgap energy decreasing from 3.11 eV (pristine SFO) to 2.29 eV (SPG nanocomposite). The SPG nanocomposite achieved a SY degradation of 93.56% under visible light and 97.28% under visible light/H_2_O_2_ within 90 minutes at pH = 3. Kinetic studies followed a pseudo-first-order model with hydroxyl radicals (·OH) identified as the dominant oxidative species and photogenerated holes (h^+^) as the secondary species. The photocatalyst retained substantial stability and reusability over three cycles. Cyclic voltammetry (CV) analysis further confirmed superior interfacial charge transport and higher peak currents. Compared with reported ferrite-based SY systems, the SPG nanocomposite delivers high degradation under visible light, with and without H_2_O_2_, highlighting its potential for the removal of anionic sulfonated azo dyes.

## Abstract 2

The newly developed SrFe_12_O_19_/PANI/Graphene nanoplatelet (SPG) nanocomposite showed efficient sunset yellow degradation under visible light/H_2_O_2_. Its highly pH-dependent activity arises from enhanced charge separation and ·OH/h^+^ radical generation.

## Graphical abstract

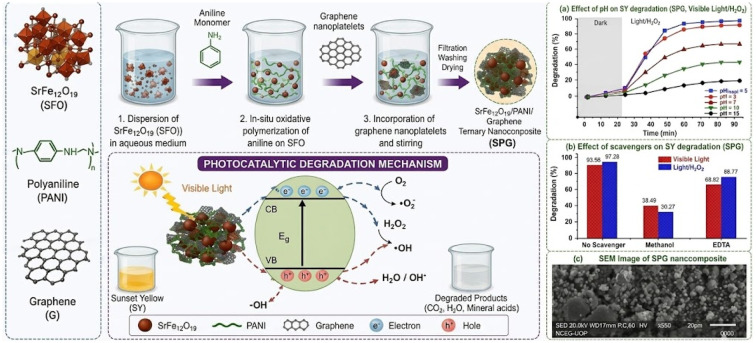

## 1. Introduction

Water pollution from synthetic dyes is a growing global problem. Industries such as textiles, leather and plastics discharge large volumes of dye-laden effluent into natural water bodies. Synthetic azo dyes alone account for ∼70% of synthetic dyes used worldwide.^1^ Among these, sunset yellow (SY) is a sulfonated monoazo dye extensively used in the food processing and textile sectors.^2^ Its highly conjugated aromatic structure and the presence of sulfonate groups make it resistant to various conventional biological treatments.^3^ Even at a concentration of 10 mg L^−1^, SY has been shown to cause oxidative stress in aquatic organisms and pose a potential human health hazard.^4,5^

Among the advanced oxidation processes (AOPs) studied for the remediation of dyes, heterogeneous photocatalysis has been widely used as an energy-efficient route for the degradation of organic pollutants. The process relies on the generation of reactive oxygen species (ROS), such as hydroxyl radicals (·OH) and superoxide anions (O_2_˙^−^), to decompose organic compounds into CO_2_, H_2_O, and other inorganic ions without producing secondary pollutants.^6^ Traditional single-phase photocatalytic systems, like ZnO and TiO_2_, despite their promise, are restricted by two well-known limitations. First, their wide bandgap (>3.0 eV) confines their photoactivation to the ultraviolet region of the solar spectrum. Second, their high recombination rates of photogenerated electron–hole pairs result in reduced quantum efficiency.^7,8^

Spinel and hexagonal ferrite semiconductors have attracted considerable research interest as photocatalysts because their narrow-to-moderate bandgaps permit visible-light excitation, while the intrinsic Fe^3+^/Fe^2+^ redox couple facilitates Fenton-type activation of H_2_O_2_ to generate additional ·OH.^9^ Among M-ferrites, strontium hexaferrite (SrFe_12_O_19_, SFO) possesses a hexagonal magnetoplumbite structure (space group *P*_63_/*mmc*) with strong intrinsic ferrimagnetism and chemical stability.^10^ Several groups have confirmed the photocatalytic activity of SFO toward different dyes. Elanthamilan *et al.*^11^ demonstrated 90.2% photocatalytic degradation of cationic methylene blue using co-precipitated SFO microspheres under visible-light irradiation, attributing the enhanced activity to the hexaferrite's planar morphology and high surface area. Irshad *et al.*^12^ achieved 91% degradation of crystal violet in 90 min by tuning the optical bandgap of Ni-doped SrNi_*x*_Fe_12−*x*_O_19_ to 1.66 eV. More recently, Ag-doped SFO synthesized by Ganguly *et al.*^13^ reached 93.1% removal of anionic Congo red in 180 min under visible light. Despite this progress, pristine SFO exhibits a relatively wide optical bandgap (2.8–3.4 eV for hydrothermally prepared samples) and high electron–hole recombination rates, both of which limit its photocatalytic efficiency under solar irradiation.^14^

Polyaniline (PANI) has consequently been investigated as a sensitizing interlayer in M-ferrite composite photocatalysts. PANI is a conducting polymer with a π-conjugated structure and a tunable bandgap. Its reversible redox transitions between leucoemeraldine and emeraldine oxidation states extend visible-light harvesting through polaronic transitions, while providing supplementary charge-transfer pathways at semiconductor interfaces.^15,16^ Shayista *et al.*^17^ investigated PANI/CuFe_2_O_4_ nanohybrids for tetracycline hydrochloride degradation and demonstrated that the optimized nanohybrid achieved 86% removal in 120 min, with the bandgap narrowing to ∼1.31 eV against 2.74 eV for bare CuFe_2_O_4_. In another study, Bashar *et al.*^18^ synthesized polyaniline-functionalized cobalt-substituted zinc ferrite (Co_1−*x*_ZnxFe_2_O_4_@PANI) and applied it to the visible-light degradation of reactive dyes.

Two-dimensional (2D) sp^2^-hybridized graphene-based materials have also been recently explored in ferrite-based photocatalysis. Unlike PANI, which primarily extends light absorption, graphene nanoplatelets (GnPs) function as high-conductivity electron-transport mediators that suppress electron–hole recombination by rapidly shuttling photogenerated electrons away from active sites.^19^ The Ni_0.5_Co_0.25_Mg_0.25_Ho_0.03_Fe_1.97_O_4_/GNPs composites synthesized by Muneer *et al.*^20^ demonstrated 88% degradation of anionic direct red 23 dye in 120 min. Similarly, Zhang *et al.*^21^ achieved 94% removal of Rhodamine B in 80 min using reduced graphene oxide-modified BiOCl/Co-doped SFO composite. More notably, Ahmad *et al.*^22^ developed a Z-scheme ternary heterostructure of graphene oxide-mediated PANI with α-Fe_2_O_3_ that achieved 99.8% degradation of brilliant green in just 25 min under visible light.

Despite these advances, ternary integration of SFO with PANI and GnPs as a visible-light and visible-light/H_2_O_2_ photocatalyst remains unreported. More critically, no prior work has targeted an anionic sulfonated azo dye like SY, with established aquatic ecotoxicity and human genotoxic concerns, using such a heterostructured photocatalytic system. The mechanistic interplay between surface charge dynamics, visible-light harvesting, and radical-mediated oxidation pathways in this ternary architecture warrants systematic investigation.

The present study therefore reports the synthesis and physicochemical characterization of a novel ternary SrFe_12_O_19_/PANI/GnPs (SPG) nanocomposite and evaluates its photocatalytic activity toward the degradation of anionic SY dye under visible-light and visible-light/H_2_O_2_ irradiation. The influence of key operational parameters, including solution pH, catalyst dosage, and initial dye concentration, on degradation efficiency is systematically investigated. Radical scavenging experiments are employed to explain the operative degradation mechanism, and photocatalytic reusability is assessed over successive reaction cycles. Electrochemical characterization by cyclic voltammetry is further performed to probe the interfacial charge-transport characteristics of the ternary system relative to pristine SFO.

## 2. Experimental details

### 2.1. Materials and methods

Strontium nitrate (Sr(NO_3_)_2_), iron nitrate nonahydrate (Fe(NO_3_)_3_·9H_2_O), and ethanol were procured from Scharlab. Aniline monomer (C_6_H_5_NH_2_) and ammonium persulfate ((NH_4_)_2_S_2_O_8_, APS) were obtained from DaeJung Chemicals and Metals, Korea. Graphite nanoplatelets (GnPs) were supplied by Asbury Carbon, USA. Sigma-Aldrich supplied hydrochloric acid (HCl, 37%), sodium hydroxide (NaOH), and hydrogen peroxide (H_2_O_2_, 30%). Sunset Yellow dye was chosen as the model anionic azo dye pollutant for assessing photodegradation performance. All aqueous solutions were prepared exclusively with ultra-pure water procured from a Milli-Q purification unit.

### 2.2. Synthesis of strontium hexaferrite nanoparticles

SFO NPs were prepared using a one-pot hydrothermal route.^23^ Precisely weighed amounts of Sr(NO_3_)_2_ and Fe(NO_3_)_3_·9H_2_O (molar ratio of Sr : Fe = 1 : 12) were co-dissolved in a 1 : 1 (v/v) binary solvent system of ethanol and distilled water under constant stirring. The solution pH was subsequently adjusted to 13 by controlled dropwise addition of 5 M NaOH. The resulting alkaline suspension was sealed in a Teflon-lined autoclave and thermally treated at 190 °C for 20 h. After cooling to room temperature, the dark-brown precipitate was collected by vacuum filtration and washed repeatedly with deionized water to remove ionic impurities. Finally, the nanoparticles were obtained after drying the precipitate at 70 °C for 10 h.

### 2.3. Synthesis of polyaniline

PANI was synthesized by chemical oxidative polymerization of aniline in an acidic medium.^24^ Aniline (0.25 mL) was dissolved in 25 mL of 0.5 M HCl under continuous stirring to prepare the protonated monomer solution. Separately, APS (0.085 M) was dissolved in 5 mL of distilled water and added dropwise to the monomer solution maintained at 5 °C. Polymerization was allowed to proceed for 5 h under vigorous stirring, yielding a dark green precipitate characteristic of the conducting emeraldine salt form of PANI. The precipitate was then collected *via* vacuum filtration using nylon filter paper and washed repeatedly with HCl and distilled water to remove impurities. Lastly, the purified product was subjected to thermal drying at 60 °C for 24 h and crushed into a uniform fine powder.

### 2.4. Synthesis of strontium hexaferrite/polyaniline/graphite nanocomposite

The SPG ternary nanocomposite was synthesized *via* the *in situ* chemical oxidative polymerization technique.^25^ Pre-synthesized SFO NPs and GnPs were dispersed into an aniline-HCl monomer solution in an ice bath at 4 °C under constant stirring. Once the reaction temperature stabilized at 4 °C, a calculated amount of APS oxidant was gradually introduced to initiate *in situ* polymerization. The mixture was then maintained under constant mechanical stirring at 4 °C for 5 h. The resultant ternary composite was thereafter collected through repeated washing cycles.

## 3. Characterization

Phase purity and crystal structure were examined by powder XRD (Bruker D8 Advance, Cu Kα, *λ* = 1.5418 Å, 2*θ* = 10°–80°). Surface morphology and elemental composition were examined by a scanning electron microscope (SEM; JSM 5910). Functional groups in the synthesized materials were identified using an Agilent Tech Cary630 FTIR spectrometer over 400–4000 cm^−1^. Optical properties were investigated by LAMBDA 365+ UV-vis diffuse reflectance spectroscopy (DRS) in the 200–800 nm wavelength range. N_2_ adsorption–desorption isotherms at 77 K were used to determine the Brunauer–Emmett Teller (BET) specific surface area, with specimens degassed at 2 mmHg pressure and 130 °C for 4 h before examination. Pore size distribution was determined by the Barrett–Joyner–Halenda (BJH) method. The point of zero charge (pH_pzc_) was determined following a similar procedure described in a prior study.^26^ Electrochemical properties were assessed by cyclic voltammetry (CV) in 0.1 M potassium ferricyanide K_3_[Fe(CN)_6_] electrolyte over the potential window of −1.0 to +0.7 V.

## 4. Photocatalytic experiments

The photocatalytic efficiency of the prepared SFO NPs and SPG ternary nanocomposite was assessed by examining the degradation of the SY dye under visible light and visible-light/H_2_O_2_ irradiation.

Photocatalytic experiments were conducted in a 500 mL cylindrical borosilicate glass immersion-well reactor under illumination from a Philips Master PL-L 55 W/840 bulb lamp positioned 15 cm above the reaction surface. It has a wavelength range of 400–700 nm with a measured irradiance of 0.95 mW cm^−2^. An external water-cooling jacket was used to maintain the reaction temperature at 25 °C ± 2 °C. For each investigation, a designated 5–20 mg of the photocatalyst was dispersed in 100 ppm of the SY solution, with a primary concentration range of 4 ppm to 20 ppm and pH levels changing between 3 and 13. Prior to illumination, the suspension was maintained under magnetic stirring in the dark for 30 min to attain adsorption–desorption equilibrium between the catalyst and the SY solution. In H_2_O_2_-assisted tests, a calculated volume of 30 wt% H_2_O_2_ solution was introduced at the onset of irradiation to achieve a final concentration of 10 mM.

Aliquots of 3 mL were withdrawn every 10 min and centrifuged at 2000 rpm for 5 min to remove the suspended catalyst. The residual SY concentration in the supernatant was subsequently quantified using a UV-vis spectrophotometer at *λ*_ma*x*_ = 482 nm. The degradation efficiency of SY was calculated as per eqn (1) as follows:

1 Degradation (%) = [(*C*_o_ − *C*_*t*_)/*C*_o_] × 100

where *C*_o_ and *C*_*t*_ denote the initial and time—*t* SY concentrations, respectively.

## 5. Results and discussion

### 5.1. Characterization analysis

#### 5.1.1. Crystal structure and phase (XRD) analysis

**Fig. 1:**
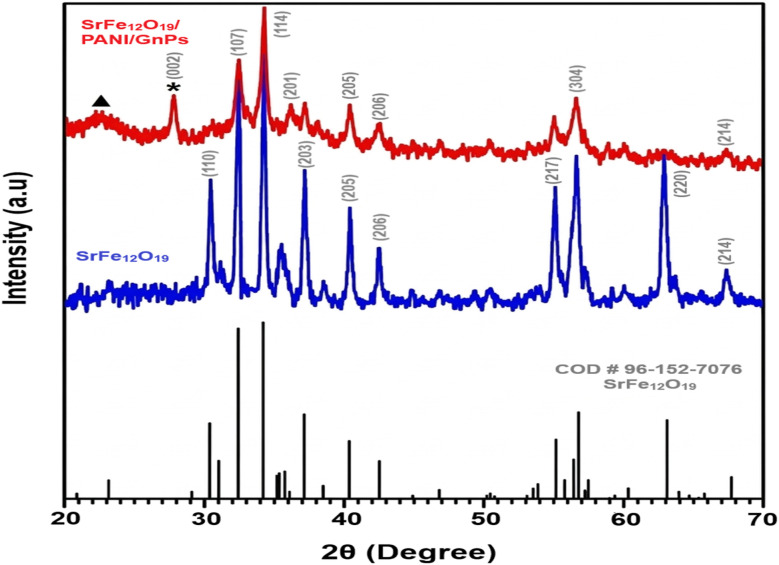
XRD patterns of the phase-pure SrFe_12_O_19_ NPs and SrFe_12_O_19_/PANI/GnPs ternary composite, referenced against the standard diffractogram of SrFe_12_O_19_ (COD #96-152-7076). The asterisk (*) denotes the GnP (002) reflection, and the triangle (▲) indicates the amorphous hump of PANI.

The XRD patterns of pristine SFO and the ternary SPG composite are presented in Fig. 1. The diffractogram of pure SFO displayed sharp, well-defined reflections at 2*θ* = 30.34°, 32.34°, 34.16°, 35.44°, 40.32°, 43.06°, 55.26°, 56.42°, 63.24°, and 67.86°, corresponding to (110), (107), (114), (201), (205), (206), (217), (304), (220), (214) planes and *d*-spacing = 2.946 Å, 2.768 Å, 2.625 Å, 2.533 Å, 2.237 Å, 2.101 Å, 1.662 Å, 1.631 Å, 1.470 Å, 1.381 Å, respectively. All diffraction peaks were indexed to the M-type hexaferrite magnetoplumbite structure (COD #96-152-7076, space group *P*_63_/*mmc*). No secondary phases from α-Fe_2_O_3_ or SrO were detected. In the SPG nanocomposite, all characteristic hexaferrite reflections were preserved with only marginal peak broadening (FWHM 0.34° at (110)), indicating that neither PANI polymerization nor GnP incorporation disrupted the crystallographic integrity of SFO NPs. A new diffraction peak at 2*θ* = 26.52°, corresponding to the (002) interlayer reflection of graphitic carbon with *d*_002_ = 3.36 Å, confirmed the structural incorporation of GnPs within the nanocomposite structure.^27^ A broad hump evident at 2*θ* = 22.06° is consistent with the amorphous character of PANI in its emeraldine salt form.^28^

The crystallite sizes of pristine SFO and SPG ternary nanocomposite were estimated from the most intense (114) reflection using the Scherrer equation displayed in eqn (2) as follows:

2 *D* = *Kλ*/*β* cos *θ*

where *k* = 0.94, *λ* = 1.5418 Å, *β* = full width at half maximum (FWHM) and *θ* = incident X-ray angle. The crystallite sizes of pristine SFO and the SPG ternary nanocomposite were calculated to be 29.45 nm and 26.27 nm, respectively. These values are nearly identical to the values calculated in previous studies.^29,30^

#### 5.1.2. Surface morphology and elemental composition (SEM-EDX) analysis

**Fig. 2:**
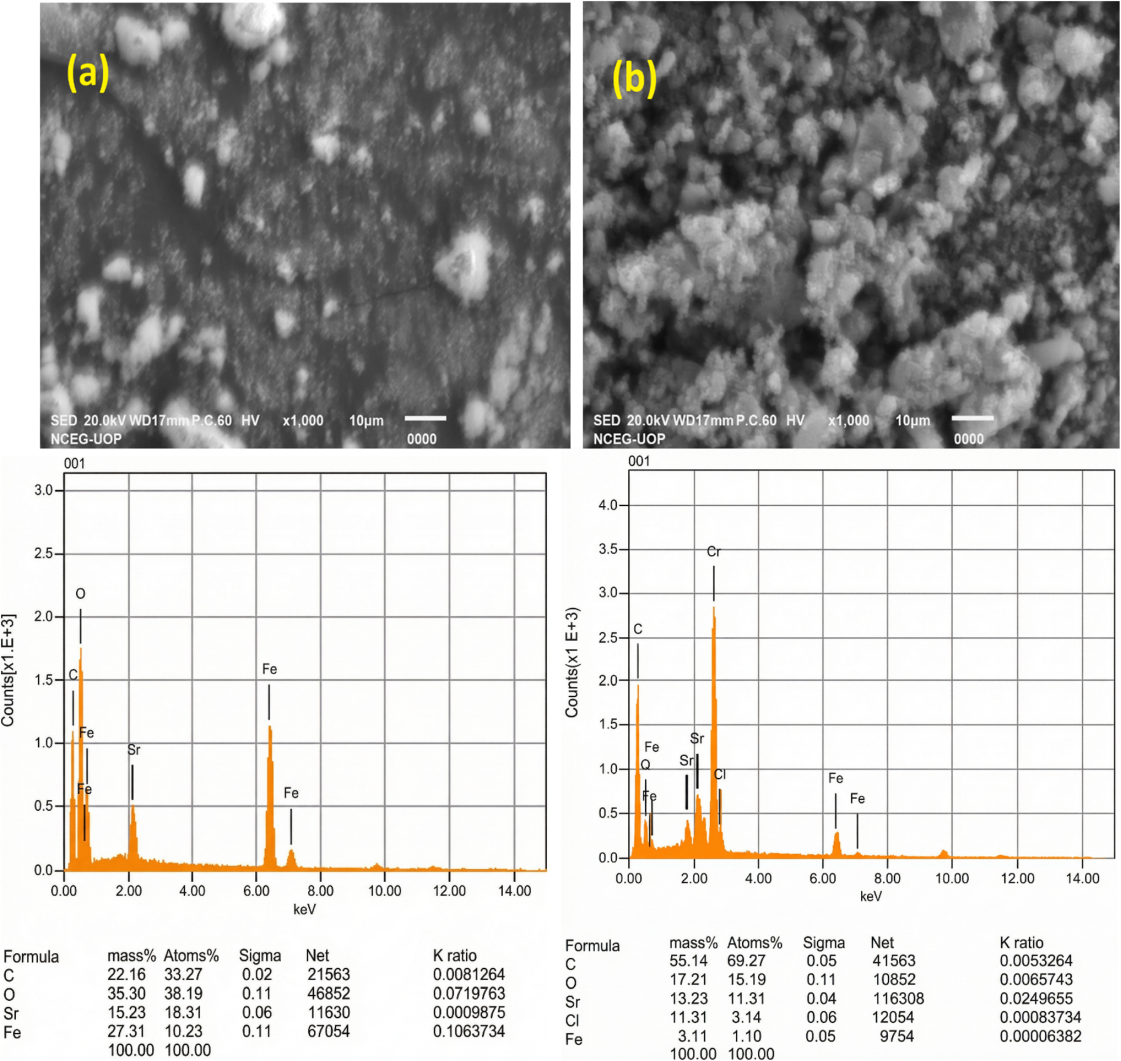
SEM images and EDX spectra of the (a) SrFe_12_O_19_ NPs and (b) SrFe_12_O_19_/PANI/GnPs.

Fig. 2a and b depicts the surface microstructure of pure SFO and SPG nanocomposite. The SEM micrograph of pure SFO (Fig. 2a) revealed an irregular, granular morphology characterized by agglomerated particulate clusters distributed heterogeneously across the surface. This could be attributed to the intrinsic magnetic interactions and high surface energy of the ferrite phase. The absence of the well-defined hexagonal platelet structure conventionally associated with the M-type strontium hexaferrite is attributable to the non-calcination hydrothermal synthesis route employed in this work. It is well established that the development of anisotropic, plate-like hexagonal morphology in SFO requires high-temperature calcination (typically ≥900 °C), which facilitates thermally driven crystallographic growth preferentially along the crystallographic *c*-axis.^31^ In the absence of such thermal treatment, kinetic constraints suppress anisotropic grain development, yielding particles of irregular form with reduced crystallographic order, consistent with the observations of Silva *et al.*^32^ In contrast, the SEM micrograph of the ternary SPG nanocomposite (Fig. 2b) reveals a markedly distinct microstructure, wherein the SFO particulate phase appears embedded within a continuous, relatively smooth matrix attributable to the PANI polymer coating. The presence of GNPs is evidenced by the faintly discernible lamellar regions distributed within the matrix. This intimate interfacial contact between phases confirms successful composite formation and is consistent with an effective heterojunction assembly.

EDX spectrum analysis confirmed the presence of Sr, Fe, O, and N in the prepared samples. The presence of additional Cl in the nanocomposites is due to washing with HCl during the synthesis process.

#### 5.1.3. Chemical bonding (FTIR) analysis

**Fig. 3:**
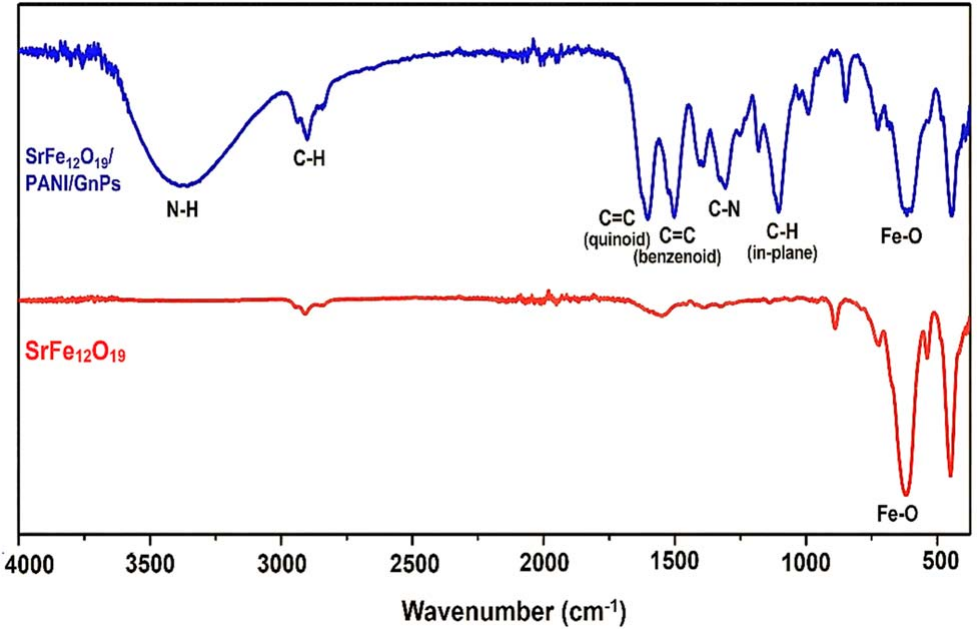
FTIR spectra of the SrFe_12_O_19_ NPs and SrFe_12_O_19_/PANI/GnPs nanocomposites.

FTIR absorption spectra of bare SFO and the SPG ternary composite are displayed in Fig. 3. The spectrum of bare SFO is dominated by Fe–O stretching vibrations in the 400–600 cm^−1^ region, characteristic of the tetrahedral and octahedral iron coordination environments in the M-type hexaferrite lattice.^33^ The weak C–H stretching bands between 3000 and 2800 cm^−1^ are most plausibly attributed to residual ethanol, which was used as a binary solvent during the synthesis process. Ethanol molecules can become physically adsorbed onto the surface of SFO particles or trapped within agglomerates during the hydrothermal process. The low intensity of the band confirms that only a negligible quantity of ethanol remained post final drying, which does not affect the structural or magnetic integrity of the SFO lattice. In the SPG nanocomposite, these Fe–O bands are retained, confirming that the hexaferrite lattice survives *in situ* polymerization conditions. The N–H stretching vibration at 3300 cm^−1^ and aromatic C–H stretching between 3000 and 2800 cm^−1^ are attributed to the presence of PANI in the nanocomposite. The C=C stretching at 1610 cm^−1^ and 1506 cm^−1^ are credited to the quinoid and benzenoid rings of the emeraldine-salt form of PANI, respectively. Whereas, the peaks at ∼1300 cm^−1^ and ∼1100 cm^−1^ are due to C–N stretching and C–H in-plane bending, respectively.^34^ The characteristic graphitic C=C ring deformation mode of GnPs appears as a shoulder near 1580 cm^−1^, partially overlapping with the PANI quinoid band.^35^ The co-occurrence of all three spectral signatures confirms the chemical co-presence of SFO, PANI, and GnPs within a single composite structure.

#### 5.1.4. Optical properties (UV-vis DRS) analysis

**Fig. 4:**
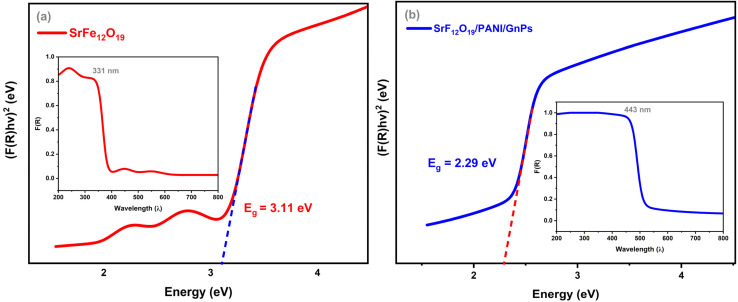
Kubelka–Munk bandgap plots of the (a) SrFe_12_O_19_ NPs and (b) SrFe_12_O_19_/PANI/GnPs nanocomposites. Insets: corresponding UV-vis DRS spectra as a function of the wavelength.

UV-vis diffuse reflectance spectroscopy (DRS) of the synthesized SFO NPs and SPG ternary nanocomposite is presented in Fig. 4. The UV-vis DRS spectrum of bare SFO NPs (Fig. 4a, inset) exhibited a sharp absorption edge near 331 nm, consistent with previously reported values for hydrothermally prepared SFO^36^ and attributable to ligand-to-metal charge transfer transitions involving O^2−^ → Fe^3+^ within the hexaferrite lattice. In contrast, the UV-vis DRS spectrum of the SPG ternary nanocomposite (Fig. 4b, inset) displayed a pronounced red-shift, with the absorption edge extending to 443 nm and persisting broadly across the visible range. This marked spectral extension is attributable to the combined contributions of PANI's π–π* benzenoid transitions and polaron–bipolaron transitions in its conducting emeraldine salt form, alongside the delocalized π-electron network of GnPs, acting as a visible-light-absorbing antenna within the heterostructure.

The bandgap values of under-examined specimens were determined from the following equation,^37^ defined as eqn (3) as follows:

3 *F*(*R*)*hν* = *A*(*hν* − *E*_g_)^2^

where *F*(*R*) is the Kubelka–Munk function, while *A* and *hν* are the proportionality coefficient and photon energy, respectively.

The Kubelka–Munk function was assessed *via* the formula given in eqn (4) as follows:

4 *F*(*R*) = (1 − *R*)^2^/2*R*

where *R* represents the diffuse reflectance.

The bandgap values of the under-examined specimens were determined by extrapolating the linear region of the [*F*(*R*)*hν*]^2^*versus hν* curve to 0. The Kubelka–Munk plot of SFO NPs yielded a direct bandgap of *E*_g_ = 3.11 eV, in line with the previous recorded values.^38,39^ Meanwhile, the SPG ternary nanocomposite displayed a reduced bandgap of 2.29 eV. This reduced bandgap was attributed to the creation of interfacial charge transfer states at the SFO-PANI heterojunction and to the visible-light sensitization capacity of the GnP network acting as a photon-absorbing antenna.

#### 5.1.5. Surface area and pore size (N_2_ adsorption–desorption) analysis

**Fig. 5:**
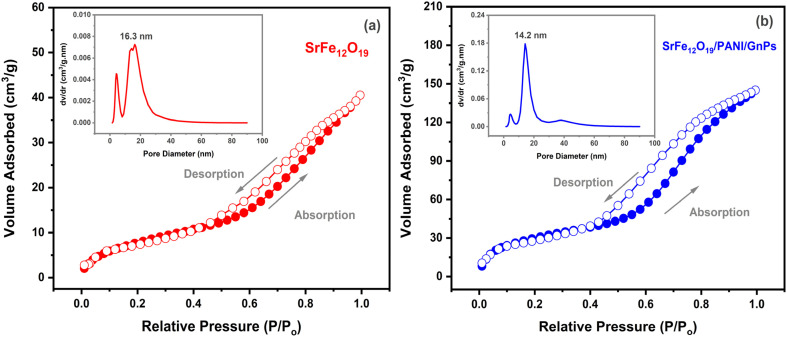
N_2_ adsorption–desorption isotherms at 77 K for the (a) SrFe_12_O_19_ NPs and (b) SrFe_12_O_19_/PANI/GnPs nanocomposites. Insets: BJH pore size distribution curves.

N_2_ adsorption–desorption isotherms at 77 K for bare SFO and the SPG ternary nanocomposite are presented in Fig. 5(a and b). Both materials exhibit Type IV isotherms with H3-type hysteresis loops in accordance with the IUPAC classification. This is indicative of mesoporous network structures in both materials.^40,41^

Based on the BET analysis, the bare SFO NPs recorded a specific surface area (SSA) and pore volume of 4.7 m^2^ g^−1^ and 0.0192 cm^3^ g^−1^, respectively, with a BJH average pore diameter of 16.3 nm. On the contrary, the ternary nanocomposite exhibited a significantly higher SSA of 16.9 m^2^ g^−1^. The corresponding pore volume and average pore diameter were calculated to be 0.060 cm^3^ g^−1^ and 14.2 nm, respectively. This enhancement in SSA is attributed to the incorporation of the high-surface-area GnPs and to the steric stabilization of SFO NPs by the PANI matrix.^42^ This enhanced SSA is expected to improve dye adsorption at the catalyst surface and increase the number of photocatalytically active sites.

#### 5.1.6. Cyclic voltammetry analysis

**Fig. 6:**
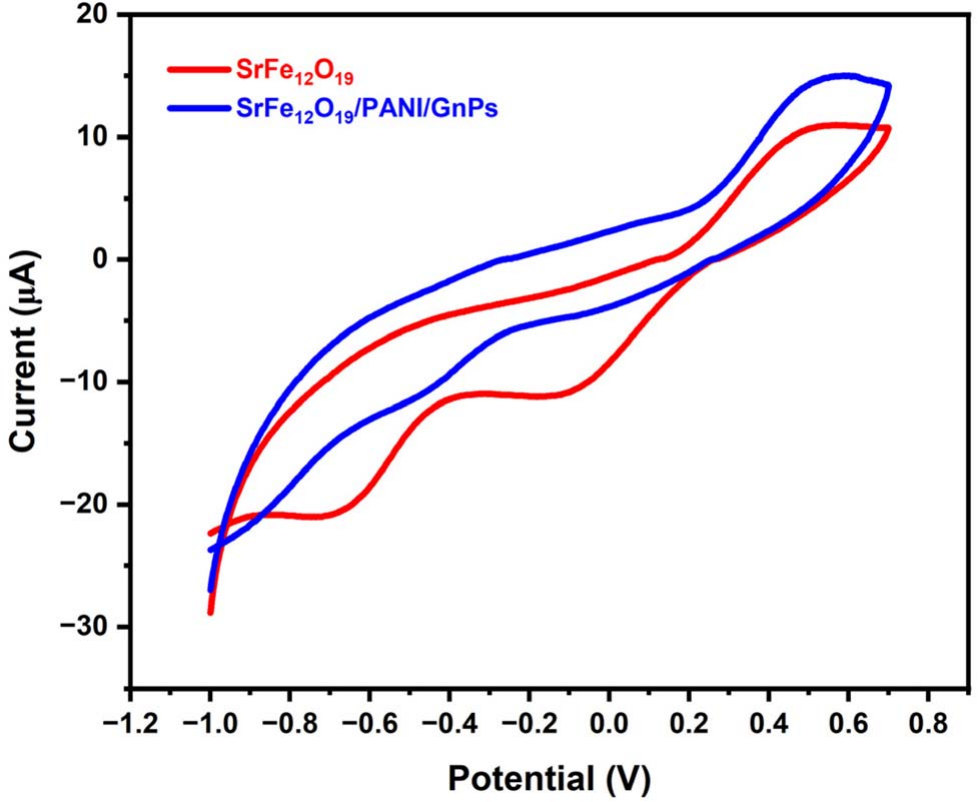
Cyclic voltammograms of SrFe_12_O_19_ and SrFe_12_O_19_/PANI/GnPs recorded in a 0.1 M potassium ferricyanide electrolyte over the potential window of −1.0 V to 0.7 V.

Cyclic voltammetry (CV) was conducted to compare the electrochemical properties of bare SFO NPs and the SPG ternary nanocomposite. Fig. 6 shows the cyclic voltammograms of under-examined samples recorded in 0.1 M potassium ferricyanide over a −1.0 to +0.7 V window. Compared to pristine SFO NPs, the ternary nanocomposite displayed significantly high peak currents with well-defined and highly symmetric redox peaks. This superior behavior is ascribed to various synergistic effects. The GnPs provided a suitable matrix for excellent electron mobility and a large specific surface area. The pseudocapacitive characteristics of PANI allowed reversible protonation and deprotonation of the matrix during potential scanning. While SFO NPs are known to have low conductivity, their incorporation into the conductive PANI/GnPs matrix helped preserve their structural integrity and provided a catalyst recovery mechanism due to its magnetic characteristics.^43^

### 5.2. Photocatalytic degradation analysis

The photocatalytic activity of the synthesized samples was evaluated under visible light, H_2_O_2_, and light/H_2_O_2_ by varying the working parameters, including pH (3 to 13), photocatalyst dosage (5 to 20 mg), and primary SY concentration (4 to 20 ppm). All experiments were performed at 298 K.

**Fig. 7:**
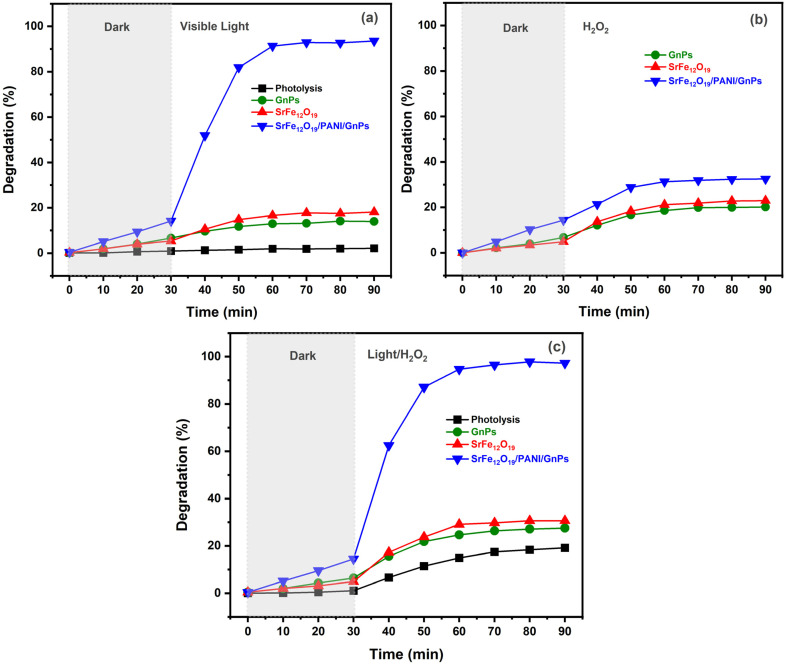
Preliminary tests and photocatalytic degradation of the SY dye under (a) visible light only, (b) H_2_O_2_ alone, and (c) visible light/H_2_O_2_ (conditions: initial pH of the solution = 3, *m*_catalyst_ = 10 mg, and SY_conc_ = 4 ppm).

Before optimizing the operational parameters, initial control experiments were conducted to assess SY photolysis and its adsorption onto pristine SFO NPs, PANI, GnPs, and the SPG ternary nanocomposite. The photocatalytic degradation of SY in the absence and presence of the catalyst under visible light, H_2_O_2_, and light/H_2_O_2_ using identical conditions (initial pH = 3 and SY_conc_ = 4 ppm) is presented in Fig. 7. As illustrated in Fig. 7a–c, the degradation of SY was negligible under all illumination conditions, reflecting its strong structural stability against direct photolysis.

To evaluate the adsorption contribution to SY degradation, adsorption tests were carried out in the dark using the pristine SFO NPs, PANI, GnPs, and the SPG ternary nanocomposite photocatalysts. The experiments were performed at an initial solution pH = 3, catalyst dosage (m_dosage_) = 10 mg, and SY_conc_ = 4 ppm. After 30 min in the dark, ∼6.6% and ∼5% of SY were adsorbed by pristine SFO NPs and GnPs. In contrast, the SPG ternary nanocomposite adsorbed 14.34% of SY. Among the tested samples, the SPG ternary nanocomposite photocatalyst displayed enhanced photocatalytic performance compared to pure SFO and GnPs. Under light/H_2_O_2_ irradiation, the SPG nanocomposite achieved 97.28% SY degradation within 90 min (Fig. 7c), while showing 93.56% degradation under visible-light irradiation (Fig. 7a). In contrast, it showed a moderate 32.48% photocatalytic degradation when exposed to H_2_O_2_ only.

Accordingly, the photocatalyst's capability to remove SY under visible light and light/H_2_O_2_ irradiations depends heavily on its bandgap characteristics. SFO NPs having a high bandgap (3.11 eV) demonstrated poor response to visible-light radiation due to the low efficiency of absorbing photons above ultraviolet. Incorporation of PANI and GnPs into the ternary SPG nanocomposite narrowed the effective bandgap (2.29 eV) and thus enabled broad light absorption in the visible region. This reduction was attributed to interfacial charge-transfer states at the SFO-PANI heterojunction and the π-conjugated GnP network, which collectively suppressed electron–hole recombination and promoted the generation of reactive oxygen species (*e.g.*, hydroxyl radicals). In addition, the increase in specific surface area (from 4.7 to 16.9 m^2^ g^−1^) further contributed to an elevated photocatalytic activity of the ternary nanocomposite.^44–46^ Based on these results, the SPG nanocomposite was selected for all subsequent parametric optimization studies.

**Fig. 8:**
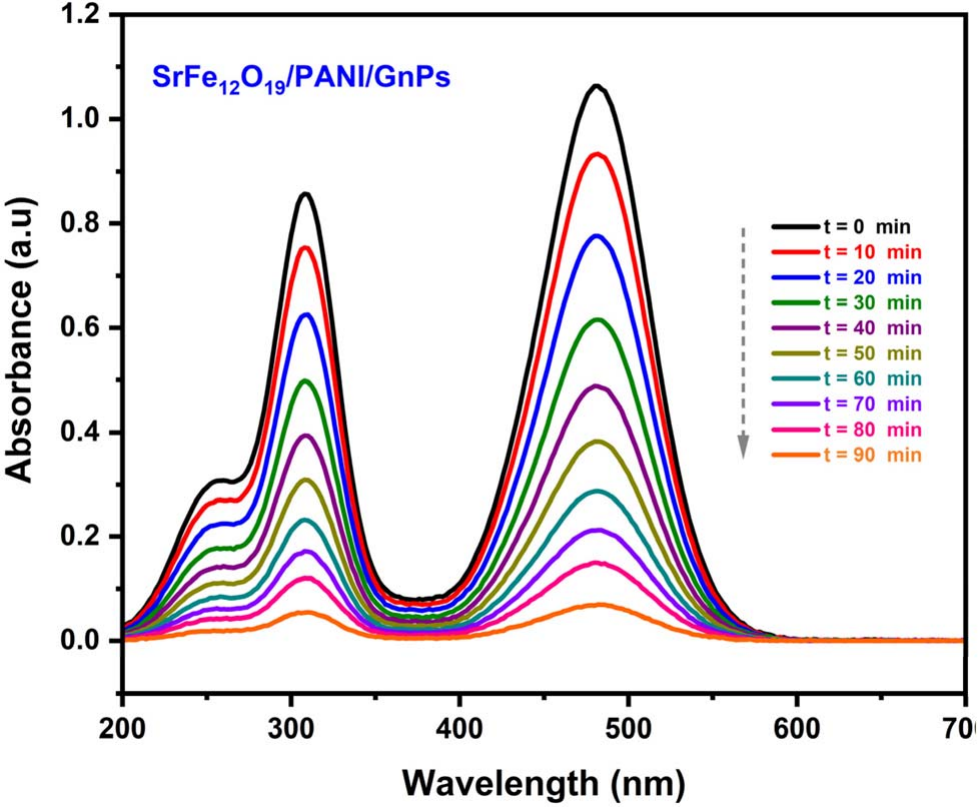
Time-dependent UV-vis absorption spectra of the sunset yellow dye during photocatalytic degradation over SrFe_12_O_19_/PANI/GnPs under light/H_2_O_2_ (conditions: initial pH of the solution = 3, *m*_catalyst_ = 10 mg, and SY_conc_ = 4 ppm).

To further verify the degradation process, UV-Vis absorption spectra of Sunset Yellow were recorded at different irradiation times under optimized conditions (initial pH = 3, *m*_catalyst_ = 10 mg, and SY_conc_ = 4 ppm). The corresponding spectra are presented in Fig. 8. The characteristic absorption band centered at ∼482 nm gradually decreased with increasing irradiation time. This band is associated with the azo chromophore (–N=N–) responsible for the visible color of SY. Simultaneously, the absorption band in the UV region near 310 nm also exhibited a continuous decrease, indicating progressive degradation of the aromatic conjugated structure of the dye molecule.

The gradual reduction of both absorption bands confirms the continuous consumption of SY during the photocatalytic reaction. After 90 min of irradiation, only a small residual absorbance remained, which is consistent with the high degradation efficiency obtained under the optimized conditions. Furthermore, no additional absorption bands were observed in the visible region during the degradation process. This suggests that colored intermediate species did not accumulate in significant amounts throughout the reaction. The observed spectral evolution indicates the effective destruction of the chromophoric structure of SY and supports the proposed hydroxyl radical-mediated oxidation pathway discussed in the section on mechanism.

#### 5.2.1. Effect of solution pH on SY degradation

**Fig. 9:**
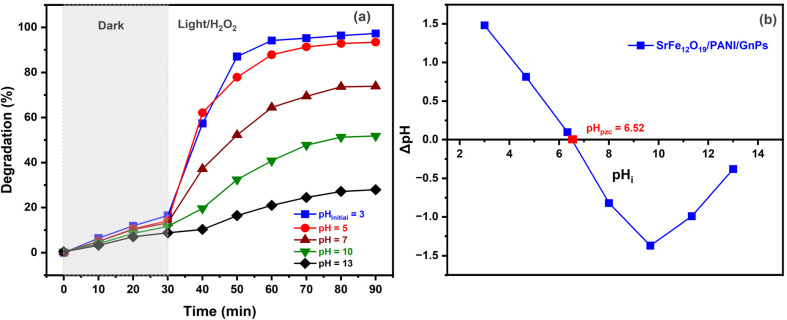
(a) Effect of pH on the photocatalytic degradation of SY with the SrFe_12_O_19_/PANI/GnPs catalyst (conditions: *m*_catalyst_ = 10 mg and SY_conc_ = 4 ppm). (b) pH_pzc_ of the SrFe_12_O_19_/PANI/GnPs catalyst.

The pH value is one of the key operational parameters that considerably affects the photocatalytic degradation of pollutants by altering the surface charge properties of the photocatalyst.^47^ The extent of this influence is generally governed by both the chemical nature of the target pollutant and the pH_pzc_ of the photocatalytic material employed.^48^ The role of pH in SY photodegradation was evaluated by running tests across the 3–13 pH range. The solution pH was adjusted with 0.1 M NaOH or HCl. In each experimental trial, 10 mg of the SPG nanocomposite catalyst was utilized to treat a 4 ppm SY solution under light/H_2_O_2_ irradiation. The corresponding results are presented in Fig. 9a.

The pH_pzc_ of the SrFe_12_O_19_/PANI/GnPs nanocomposite was calculated to be 6.52, as presented in Fig. 9b. At pH < pH_pzc_, the catalyst surface carries a net positive charge, whereas at pH levels above pH_pzc_, the surface is negatively charged. SY carries two sulfonate functional groups with p*K*_a_ < 1, which causes the dye to act as a dianion throughout the entire tested pH range.^49,50^ Consequently, the maximum electrostatic attraction between the positively charged catalyst surface and the anionic SY molecule is expected under acidic conditions.

As evident in Fig. 9a, the highest degradation was observed at pH = 3 due to strong interactions between the positively charged catalyst surface and the anionic SY dianion. This facilitated enhanced interfacial adsorption and accelerated chromophore oxidation. At pH = 5, a comparatively diminished degradation efficiency was noted as the narrowed pH–pH_pzc_ gap reduced surface charge density and progressively weakened the electrostatic driving force for the dye-catalyst interaction. As the pH increased from 3 to 7 to 10 and 13, the degradation percentage dropped from 97.28% to 73.83% to 51.75% and 27.92%, respectively. At pH values exceeding pH_pzc_, the negatively charged catalyst surface repelled the anionic SY dianion, limiting the dye-catalyst interfacial contact and reducing photocatalytic efficiency. These results are in line with the findings of previous studies.^51,52^

#### 5.2.2. Effect of catalyst dosage on SY degradation

**Fig. 10:**
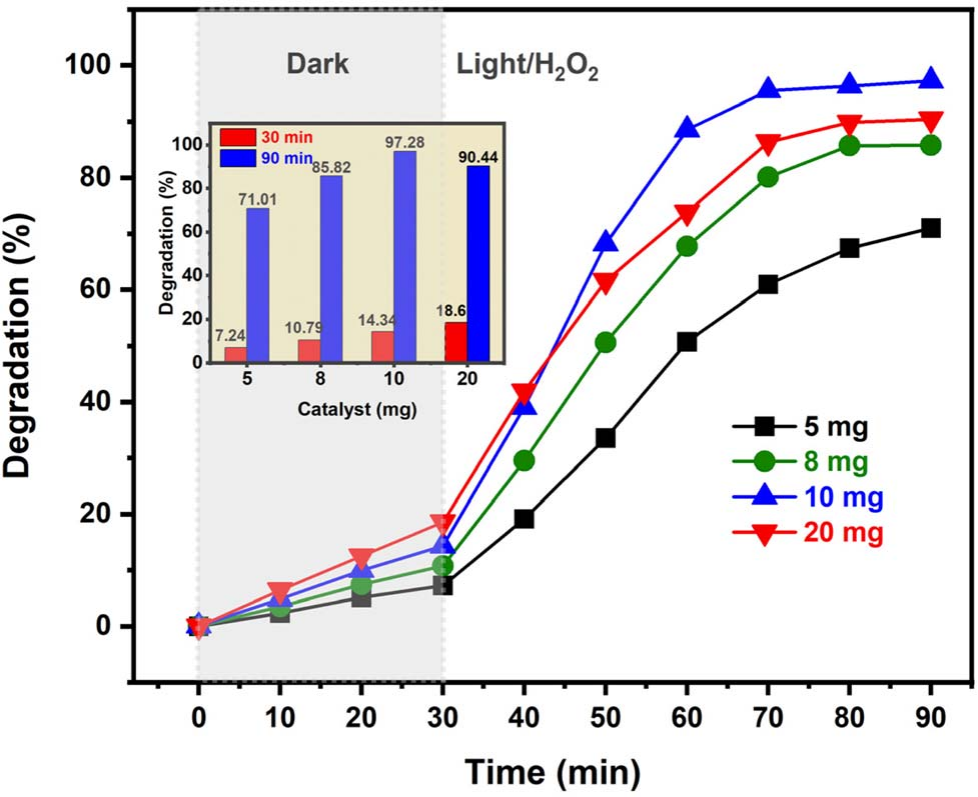
Effect of catalyst dosage on the SY degradation using the SrFe_12_O_19_/PANI/GnPs catalyst (conditions: initial pH of the solution = 3 and SY_conc_ = 4 ppm).

To determine the optimal catalyst dosage for maximum photocatalytic degradation, experiments were conducted at doses of 5, 8, 10, and 20 mg at initial pH = 3 and SY_conc_ = 4 ppm. As illustrated in Fig. 10, increasing the catalyst dosage from 5 mg to 10 mg improved the degradation efficiency. This is due to the availability of more active sites for better adsorption of light/H_2_O_2_, resulting in the generation of more ROS.^47^ However, beyond the optimal dosage, the degradation percentage decreased upon increasing the catalyst loading to 20 mg. Higher catalyst loading leads to increased turbidity and light scattering by excess catalyst particles, reducing the penetration depth of incident visible light into the reaction solution (shielding effect).^46^ This limits photon availability for photoexcitation of the catalyst, consequently reducing ROS generation and degradation efficiency.^47^ Additionally, particle agglomeration at higher loadings reduces the effective surface area exposed to incident light. Thus, a catalyst loading of 10 mg was selected as the optimal value for all subsequent photocatalytic experiments.

#### 5.2.3. Effect of dye concentration on degradation

**Fig. 11:**
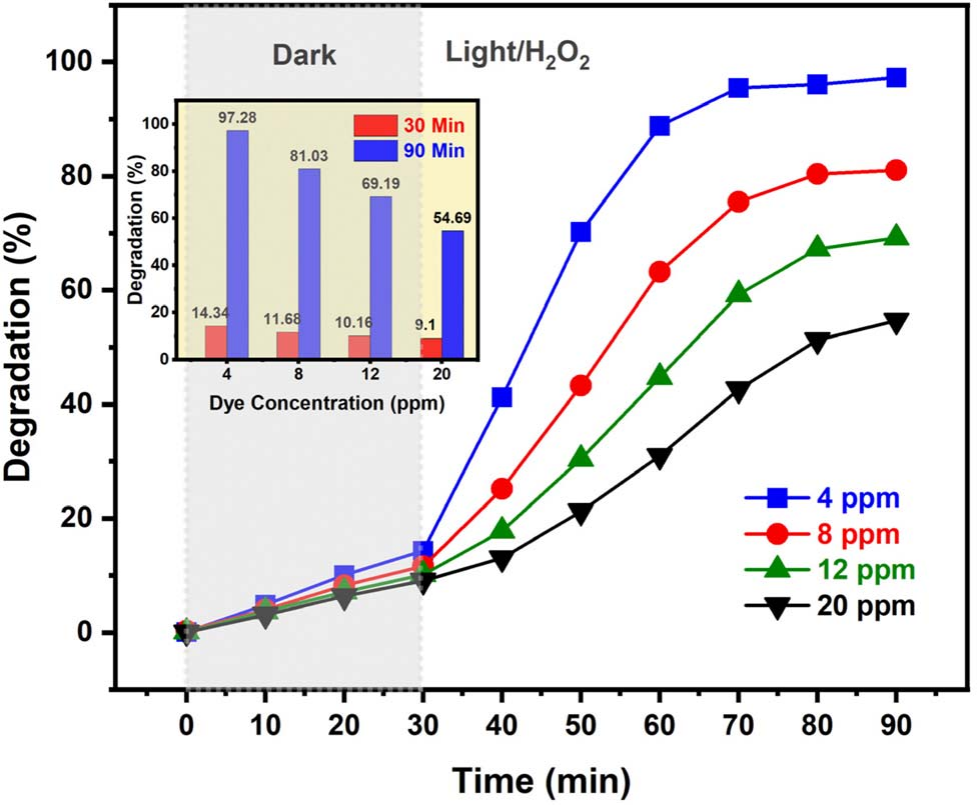
Effect of initial concentration of the dye solution on the photocatalytic degradation of SY using the SrFe_12_O_19_/PANI/GnPs catalyst (conditions: initial pH of the solution = 3 and *m*_catalyst_ = 10 mg).

The effect of initial SY concentration on photocatalytic degradation efficiency is presented in Fig. 11. It was evaluated by varying the initial SY concentration at optimal pH = 3 and *m*_catalyst_ = 10 mg. The results demonstrate that initial dye concentration exerts a considerable influence on the photodegradation process. Specifically, increasing the SY concentration from 4 to 8, 12, and 20 ppm led to a progressive decline in degradation efficiency from 97.28% to 81.03%, 69.19%, and 54.69%, respectively. This inverse relationship can be ascribed to active site saturation at elevated dye concentrations, which limits photon absorption and ROS generation.^47^ Furthermore, dye molecules absorb a larger portion of the incident light before it reaches the catalyst surface, reducing photon availability for the photocatalytic reaction (inner filter effect).^53^

#### 5.2.4. Reusability of the photocatalyst and effect of scavengers

**Fig. 12:**
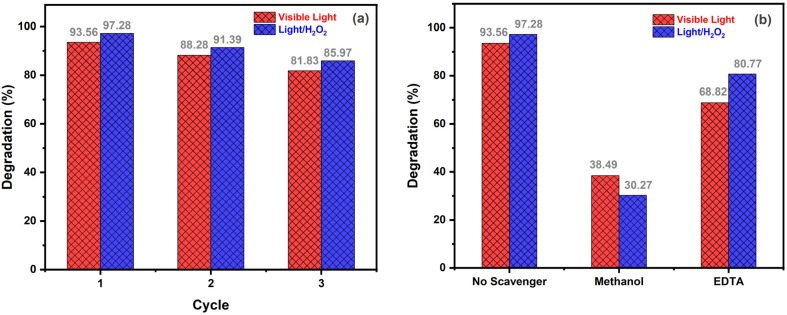
(a) Reusability performance and (b) effect of scavengers on the photodegradation of SY using the SrFe_12_O_19_/PANI/GnPs catalyst under both visible light and light/H_2_O_2_ irradiation (conditions: initial pH of the solution = 3, *m*_catalyst_ = 10 mg and SY_conc_ = 4 ppm).

The photocatalytic reusability of the SPG ternary nanocomposite was assessed over three successive cycles under both visible light and light/H_2_O_2_ at optimal experimental parameters. After each cycle, the catalyst was washed with distilled water, filtered, and dried at 90 °C prior to the subsequent run. As presented in Fig. 12a, degradation efficiency under visible light decreased gradually from 93.56% to 81.83% over three cycles. Under light/H_2_O_2_, a similar trend was observed, with efficiency declining from 97.28% to 85.97% over three cycles. The non-significant reduction in performance across successive cycles can be attributed to the partial blocking of active sites by residual dye intermediates adsorbed onto the catalyst surface during prior runs. The findings demonstrate that the ternary nanocomposite catalyst maintained reasonable photostability without significant deterioration upon repeated use.

Scavenging tests were performed to identify the reactive species involved in the photocatalytic degradation of SY using the SPG ternary nanocomposite under both visible light and light/H_2_O_2_. Methanol and EDTA were employed as scavengers for hydroxyl radicals (·OH) and photogenerated holes (h^+^), respectively. The photocatalyst was introduced following the SY solution reaction with the scavenger. As presented in Fig. 12b, the introduction of scavengers on SPG ternary nanocomposite resulted in a decrease in photocatalytic degradation under light/H_2_O_2_ from 97.28% to 30.27% and 80.77% in the presence of methanol and EDTA, respectively. A similar situation was observed under visible light treatment, with the photocatalytic degradation percentage dropping from 93.56% to 38.49% and 68.82% in the presence of methanol and EDTA, respectively. The substantially greater suppression observed upon methanol addition indicates that ·OH radicals are the principal reactive species driving SY degradation under both irradiation conditions, while h^+^ plays a secondary but contributing role. Notably, the more pronounced inhibition observed under light/H_2_O_2_ conditions (*Δ* = 66.99%) relative to visible light alone (*Δ* = 55.07%) is consistent with H_2_O_2_ serving as an auxiliary source of ·OH radicals *via* photolytic cleavage (H_2_O_2_ + *hν* → 2·OH) and conduction band electron scavenging (H_2_O_2_ + e^−^ → ·OH + OH^−^). These findings confirm that the photocatalytic degradation of SY proceeds primarily through a hydroxyl radical-mediated oxidation pathway, wherein H_2_O_2_ operates as a reactive oxidant precursor under photo-Fenton-like conditions, synergistically enhancing the intrinsic photocatalytic activity of the SPG ternary nanocomposite.^54,55^

#### 5.2.5. SY photodegradation mechanism

Monitoring of SY photodegradation indicated that hydroxyl radicals (·OH) and photogenerated holes (h^+^) functioned as the main reactive species in the oxidation process. Upon exposure to visible light, the photocatalyst absorbs photons and produces electron–hole pairs according to eqn (5)^54^ as follows:

5 SrFe_12_O_19_/PANI/GnPs + *hν* → e_CB_^−^ + h_VB_^+^

In the second step, the photogenerated holes in the valence band oxidize surface-adsorbed water or hydroxide ions to produce ·OH radicals as per eqn (6)^56^ as follows:

6 h_VB_^+^ + H_2_O/OH^−^ → ·OH + H^+^

In the third step, the interaction of H_2_O_2_ with the photogenerated electrons results in more hydroxyl radicals along with OH^−^ species (eqn (7))^54^ as follows:

7 H_2_O_2_ + e^−^ → ·OH + OH^−^

In step four, the photogenerated electrons in the conduction band are likely transferred through the conductive PANI and GnPs network. This interfacial pathway appears to suppress recombination and allows electrons to interact with dissolved oxygen (eqn (8) and (9)) as follows:

8 e_CB_^−^ + O_2_ → ·O_2_^−^

9 ·O_2_^−^ + H^+^ → HO_2_·

The generated ·OH radicals subsequently attacked the chromophoric azo linkage (–N=N–) and the sulfonate functional groups of the SY molecule, initiating their breakdown into smaller intermediates and eventually mineralization to CO_2_ and H_2_O (eqn (10))^54^ as follows:.

10 SY + ·OH → degradation by-products

Under visible light/H_2_O_2_ irradiation, H_2_O_2_ contributes two additional ·OH generation pathways.^57^ Firstly, direct photolytic cleavage of H_2_O_2_ under incident illumination (eqn (11)) as follows:

11 H_2_O_2_ + *hν* → 2·OH

Secondly, conduction band electrons reduce H_2_O_2_ at the Fe^3+^/Fe^2+^ redox-active sites on the SrFe_12_O_19_ surface *via* a photo-Fenton-like route according to eqn (12)–(14):^57^

12 H_2_O_2_ + e_CB_^−^ → ·OH + OH^−^

13 Fe^3+^ + e_CB_^−^ → Fe^2+^

14 Fe^2+^ + H_2_O_2_ → Fe^3+^ + ·OH + OH^−^

**Fig. 13:**
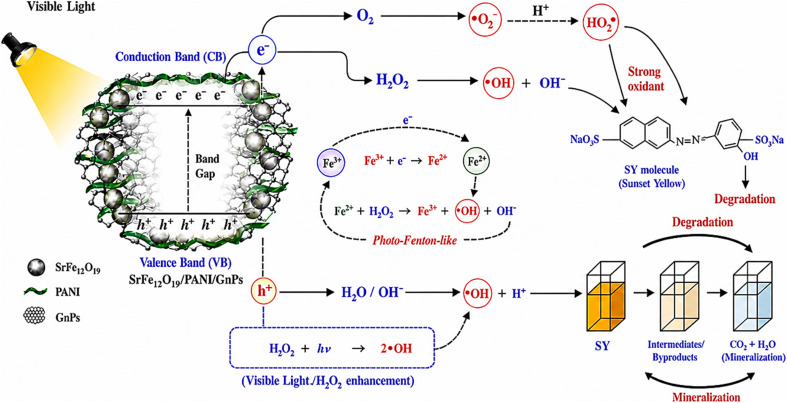
Photocatalytic mechanism under both visible-light and light/H_2_O_2_ irradiation.

This mechanism underscores the critical contributions of ·OH and h^+^ toward accomplishing effective photodegradation. The schematic of the photodegradation mechanism is depicted in Fig. 13.

#### 5.2.6. Kinetic analysis of the SY dye

The photocatalytic degradation kinetics of SY were modelled using pseudo-first-order (eqn (15)) and pseudo-second-order (eqn (16)) rate expressions as follows:

15 ln(*C*_o_/*C*_*t*_) = *K*_1_*t*

**Fig. 14:**
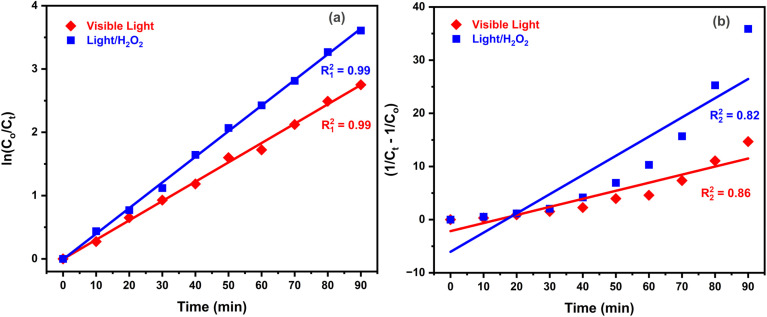
Kinetic plots of the photocatalytic degradation of SY using the SrFe_12_O_19_/PANI/GnPs catalyst under both visible-light and light/H_2_O_2_ irradiation (conditions: initial pH of the solution = 3, *m*_catalyst_ = 10 mg and SY_conc_ = 4 ppm).

16 1/*C*_*t*_ − 1/*C*_o_=*K*_2_*t*

where *C*_o_ and *C*_*t*_ represent the SY concentration at *t* = 0 and *t* = *t*, respectively, while *K*_1_ and *K*_2_ are the respective first- and second-order apparent rate constants. The corresponding plots of ln(*C*_o_/*C*_*t*_) and (1/*C*_*t*_ − 1/*C*_o_) *vs.* time are presented in Fig. 14a and b. The high correlation coefficients (*R*_1_^2^ = 0.99) confirmed that the SY photocatalytic degradation follows pseudo-first-order kinetics for both visible light and light/H_2_O_2_. Furthermore, the higher rate constant *K*_1_ obtained under light/H_2_O_2_ indicates that the additional ·OH radical flux generated *via* H_2_O_2_ photocleavage and photo-Fenton surface reactions at the Fe^3+^/Fe^2+^ sites of the SFO component accelerates the degradation rate. The pseudo-first-order kinetic behaviour is consistent with the Langmuir–Hinshelwood heterogeneous photocatalytic mechanism.^58^Table 1 shows comparative study of photocatalytic degradation of synthesized specimens with previous studies.

**Table 1:** Comparison of the photocatalytic degradation performance of the present study with previously reported ferrite, PANI, and graphene nanocomposite systems toward the degradation of anionic dyes

Catalyst	Targeted dye	pH	Catalyst dosage	Initial concentration	Reaction time (min)	Light source	Degradation (%)	Reference
SrFe_12_O_19_	Methyl orange	—	0.1 g	5 mg	220	Hg lamp (400 W and *λ* > 400 nm)	95	59
Ag–TiO_2_/Na-BNT	Sunset yellow	3	0.1 g	10 mg	120	Solar light and solar light/30% H_2_O_2_	98 and 99.9	60
CdS/MoS_2_/rGO	Sunset yellow	3	50 mg	20 ppm	180	Xenon lamp (300 W and *λ* ≧ 420)	95	61
Fe_3_O_4_/ZnO/Si_3_N_4_	Sunset yellow	—	20 mg	50 mg L^−1^	80	Xenon lamp (visible)	90	62
Fe_3_O_4_/HAP/Ag	Sunset yellow	8	2 mg	40 mg L^−1^	40	Visible light	95.6	54
Tin-doped BiFeO_3_/Graphene nanoplatelets	Congo red	—	—	100 mg L^−1^	60	Xenon lamp (300 W)	100	63
Cu/Gd-SrFe_12_O_19_/CNTs	Bromocresol blue	—	10 mg	10 ppm	60	Solar light	87.6	64
PANI/GO/MoS_2_	Methyl orange	7.6	0.2 g L^−1^	20 ppm	120	LED light	99	65
SrFe_12_O_19_–Fe_3_O_4_	Remazol red ultra RGB	4	40 mg	25 mg L^−1^	60	Mercury vapor lamps (20 W)/1 mM H_2_O_2_	100	66
SrFe_12_O_19_/PANI/GnPs	Sunset yellow	3	10 mg	4 ppm	90	Philips Master PL-L [55 W and *λ* = 482 nm] and visible light/30%H_2_O_2_	93.56 and 97.28	This work

## 6. Conclusion

The present investigation establishes that ternary integration of strontium hexaferrite with polyaniline and graphite nanoplatelets yielded a photocatalytic system with meaningfully improved visible-light activity relative to bare SFO. The synthesis of the SrFe_12_O_19_/PANI/GnPs ternary nanocomposite *via in situ* oxidative polymerization produced a structurally integrated material, as confirmed across multiple characterization techniques. XRD verified the preservation of the hexaferrite phase alongside GnP and amorphous PANI signatures, while FTIR spectroscopy confirmed chemical co-existence of all three components. Bandgap reduction from 3.11 to 2.29 eV and a 3.6-fold increase in specific surface area translated directly into 93.56% and 97.28% degradation of Sunset Yellow under visible light and light/H_2_O_2_, respectively, within 90 min. Scavenger experiments established ·OH as the dominant oxidative species, with H_2_O_2_ amplifying this pathway through photo-Fenton-like surface reactions at Fe^3+^/Fe^2+^ redox sites. Photogenerated holes served the secondary role. This finding was consistent with the pseudo-first-order kinetics observed across irradiation conditions. The catalyst retained reasonable activity over three reuse cycles, with degradation dropping by roughly 11–12%. This drop was attributable to partial active-site blockage rather than structural degradation. Electrochemical analysis supported enhanced interfacial charge transport relative to pristine SFO. These results position the SrFe_12_O_19_/PANI/GnPs nanocomposite as a viable candidate for the remediation of anionic sulfonated azo dyes.

Future studies should prioritize post-reusability characterization (*e.g.*, FT-IR and XRD) of the spent SrFe_12_O_19_/PANI/GnPs catalyst to evaluate its structural integrity after repeated cycling. Such analyses would help identify possible phase changes, polymer degradation, or delamination of graphene nanoplatelets, offering insights into deactivation mechanisms and guiding the design of more stable ternary photocatalysts. Furthermore, while the present study evaluated hydroxyl radicals and holes, the potential role of superoxide radicals (O_2_˙^−^) was not investigated. Future studies should therefore include selective superoxide scavengers (*e.g.*, *p*-benzoquinone) to complete the reactive oxygen species profile and better resolve the charge-transfer pathways in this ternary heterostructure.

## Conflicts of interest

The authors declare that they have no competing financial interests, personal relationships, or affiliations that could have influenced the results or interpretation of the work presented in this study.

## Data Availability

The data for this article, including the effect of catalyst loading on degradation, effect of initial dye concentration on degradation, kinetic plots, and bandgaps, are available at the Science Data Bank at DOI: 10.57760/sciencedb.36510.
